# Low-Dimensional Dynamics of Brain Activity Associated with Manual Acupuncture in Healthy Subjects

**DOI:** 10.3390/s21227432

**Published:** 2021-11-09

**Authors:** Xinmeng Guo, Jiang Wang

**Affiliations:** 1 School of Electrical and Information Engineering, Tianjin University, Tianjin 300072, China; jiangwang@tju.edu.cn; 2 Academy of Medical Engineering and Translational Medicine, Tianjin University, Tianjin 300072, China

**Keywords:** acupuncture, EEG, dimensionality, neural subspace, latent variables, attractor

## Abstract

Acupuncture is one of the oldest traditional medical treatments in Asian countries. However, the scientific explanation regarding the therapeutic effect of acupuncture is still unknown. The much-discussed hypothesis it that acupuncture’s effects are mediated via autonomic neural networks; nevertheless, dynamic brain activity involved in the acupuncture response has still not been elicited. In this work, we hypothesized that there exists a lower-dimensional subspace of dynamic brain activity across subjects, underpinning the brain’s response to manual acupuncture stimulation. To this end, we employed a variational auto-encoder to probe the latent variables from multichannel EEG signals associated with acupuncture stimulation at the ST36 acupoint. The experimental results demonstrate that manual acupuncture stimuli can reduce the dimensionality of brain activity, which results from the enhancement of oscillatory activity in the delta and alpha frequency bands induced by acupuncture. Moreover, it was found that large-scale brain activity could be constrained within a low-dimensional neural subspace, which is spanned by the “acupuncture mode”. In each neural subspace, the steady dynamics of the brain in response to acupuncture stimuli converge to topologically similar elliptic-shaped attractors across different subjects. The attractor morphology is closely related to the frequency of the acupuncture stimulation. These results shed light on probing the large-scale brain response to manual acupuncture stimuli.

## 1. Introduction

Acupuncture, an ancient practice in traditional Chinese medicine (TCM), is gradually being recognized throughout the world as an important modality of alternative and complementary medicine [1,2]. The World Health Organization (WHO) and the National Institutes of Health (NIH) have reported that acupuncture is an efficient treatment for various conditions, such as addiction, headaches, myofascial pain, and lower back pain [3,4,5,6]. A number of available pieces of evidence have demonstrated that acupuncture may also help with stroke rehabilitation [7]. However, the scientific explanation of acupuncture’s effects is still unknown. Clinical and experimental studies have indicated that acupuncture, as a complex somatosensory stimulation of the central nervous system, can mediate the electrical activity of autonomous neuronal networks [8,9]. Furthermore, neuroimaging data strongly suggest that widely distributed cortical and subcortical brain areas are recruited during acupuncture stimulation [10,11]. For example, Bai et al. demonstrated that acupuncture can increase activity in the amygdala, the perigenual anterior cingulate cortex (pACC), the periaqueductal gray (PAG), and the hypothalamus [12]. Therefore, more attention has been focused on probing brain activities during and after acupuncture stimulation.

In addition, an electroencephalogram (EEG) is an effective method for obtaining brain electrical signals, and is able to record spontaneous cerebral activity with a time resolution at the millisecond level. It has been widely used in clinical and experimental studies to analyze brain activity associated with acupuncture stimulation. Methods of characterizing brain activity based on EEG recordings can be divided into two categories. The first category is the statistical analysis of brain oscillatory activity, such as power spectral density, complexity, and coherence [13,14,15]. For example, Tanaka et al. investigated the variance of EEG power induced by acupuncture. They found that acupuncture could increase EEG power in all frequency bands, and this increment remained after acupuncture [16]. Furthermore, Qi et al. quantified the approximate entropy (ApEn) of EEG signals and confirmed the variance of ApEn in the prefrontal lobe, the posterior temporal lobe, and the occipital lobe before, during and after acupuncture stimulation [17]. The other category involves constructing a functional network based on various measurement of correlation or synchronization [18,19]. Yu et al. constructed the functional network of acupuncture EEG signals based on phase synchronization and found that acupuncture at ST36 can significantly improve the synchronization of alpha rhythms and enhance the small-world connection characteristics of the brain’s functional network [20,21].

Brain activity is a high-dimensional dynamical process that evolves over time, and the data analysis methods above cannot be directly associated with brain dynamics, which poses a challenge in probing the dynamic response of the brain to acupuncture stimuli. As a usual feature of complex systems, the degrees of freedom traversed by its dynamics are much lower than the number of units comprising the system [22]. The human brain is such a complex system of numerous neurons coupled through synapses. Observations in electrophysiological experiments have demonstrated that the brain has low dimensionality at different levels, from macroscopic, to the mesoscopic and microscopic scales [23,24,25]. Based on this perspective, several neuroscientists have focused on investigating the low-dimensional dynamics of brain. They suggest that a low-dimensional representation of brain, known as “latent variables”, can afford a deeper understanding of the core principles underpinning whole-brain patterns of neural activity [26,27,28]. For example, Cueva et al. found that low-dimensional dynamics provide a mechanism for the brain to solve the problem of storing information across time [29]. Abbaspourazad et al. extracted the low-dimensional dynamics in both spiking and LFP recordings within the motor cortex during reach-and-grasp tasks, and addressed that the multiscale, low-dimensional motor cortical state dynamics accounted for the neural control of motor behaviors [30].

Additionally, these latent variables are explanatory variables that are not directly observed but can be identified from the data using dimensionality reduction methods. These methods transform high-dimensional data into low-dimensional representations that retain important features of interest [31]. The variational auto-encoder (VAE) method is one of dimensionality reduction methods that consists of unsupervised neural networks, in which latent variables can be learned from the original high-dimensional datasets [32]. VAE is composed of an encoder and a decoder, the former is responsible for inferring the latent variables, and the latter is designed to generate a new dataset based on latent variables. This method shows good applicability in the study of brain activity. For example, Bi et al. put forward a semi-supervised VAE method to probe low-dimensional representations of ERPs, and found that the latent variables are of good applicability in brain-controlled vehicles [33]. Furthermore, the knowledge of low dimensional dynamics extracted from video-evoked cortical responses can predict its response with high accuracy, which has the potential to explain the cortical response scientifically [34]. Li et al. utilized VAE to learn the latent variables from the multichannel EEG signals and found that emotion recognition achieves excellent performance based on the learnt latent variables [35].

At present, study on brain activity under acupuncture stimuli mostly focus on the study of rhythm, complexity, synchronization, and functional networks. However, the brain is a high-dimensional, complex system composed of numerous neurons, and the response of the brain to acupuncture stimulation is associated with many distributed coupled cortical areas. To solve the problem of high dimensionality, we proposed to apply a dimensionality reduction method to probe the latent dynamics of brain activity associated with acupuncture stimulation. Latent variables can not only reflect the lower-dimensional features of brain activity, but can also yield clues about the underlying associated neural dynamics related to the intrinsic properties of external stimuli [36,37]. Specifically, we adopted the VAE method to extract latent variables from the experimental acupuncture signals, and further explored the brain activity associated with acupuncture stimuli.

Acupuncture is a complex interventional stimulation of the human body. Multiple stimulation parameters, including the needle sensation, acupoint specificity, acupuncture manipulation, and needle duration, have relevant influences on brain activity. Acupuncture manipulation is a key factor that determines the therapeutic effect of acupuncture. It is reported that acupuncture can reduce acute lower back pain for patients, and the improvement critically depends on the acupuncture manipulation. Therefore, this work focused on investigating the instant effect of acupuncture manipulation on brain activity. It was found that the characteristics of latent dynamics are associated with acupuncture manipulation. Overall, these results can provide a theoretical support for the selection of an appropriate acupuncture frequency for patients in clinical settings, and the proposed methods have potential in exploring the effects of acupuncture on brain activity.

This paper is organized as follows. In Section 2, the experimental acupuncture procedure and the corresponding method of analysis are introduced. In Section 3, the results are presented. Finally, the discussion and conclusion are provided in Section 4 and Section 5, respectively.

## 2. Materials and Methods

### 2.1. Experiment Design and EEG Recording

Twelve right-handed healthy subjects (7 female, 5 male, mean age 23 years, range 22–25 years), who had never been treated with acupuncture, participated in the acupuncture experiment. They confirmed that they had not been taking any medication in the past 30 days and had no history of mental illness. Participants were informed about the needle stimulation in the acupuncture experiments and gave written informed consent to participate in the experiment. The Institutional Review Board of Armed Police Logistics College Affiliated Hospital approved our experimental protocol (LLKYPJ2010005).

**Figure 1:**
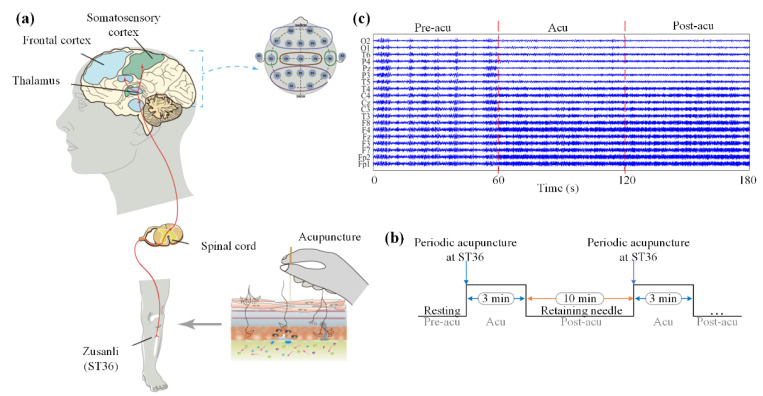
The schematic diagram of the experimental operation. (**a**) Schematic diagram of the acupuncture experiment. Electroencephalographic signals evoked by manual acupuncture at the ST36 acupoint of healthy subjects were directly recorded in three states: pre-acupuncture, acupuncture, and post-acupuncture. (**b**) A timeline of the detailed experimental procedure of manual acupuncture manipulation and (**c**) the EEG signals recorded.

In our experiment, acupuncture was administered manually at the ST36 (Zusanli) acupoint on the left leg (shown in Figure 1a) by a licensed acupuncturist using a single-use stainless steel needle of 0.2 mm in diameter and 40 mm in length. We adopt the twirling-twisting method with different frequencies as the acupuncture manipulation method. Specifically, the needle was twirled, mainly with the thumb forward, and the twisting was within a range of 90–180° and at a certain frequency. The subjects were randomly divided into three groups (four subjects in each group), which received manual acupuncture stimulation with different twirling and twisting frequencies of 50 times/min, 100 times/min, and 150 times/min, respectively.

The experiment was carried out in a dark, quiet room. The participants were asked to keep their eyes closed and stay awake to eliminate significant electromyoelectrical disturbance. For each subject, the entire experiment lasted about 59 min. The experimental procedure was carried out as follows (shown in Figure 1b): all subjects first rested for 10 min, then the acupuncture needle was inserted by the acupuncturist to a depth of 10 mm at the ST36 acupoint until deqi. The needle was kept inserted without operation for 10 min, referred to as the pre-acupuncture state (Pre-acu). Then, the twirling-twisting operation was conducted for 3 min (acupuncture, Acu). After the operation, it was necessary to keep the subject in a resting state for 10 min (post-acupuncture, Post-acu). This procedure was repeated 3 times. Finally, after removing the needle, the acupuncturist finished the experiment.

EEG signals were recorded using a Neuroscan system with 19 Ag-AgCl electrodes, which were placed in accordance with the international standard 10–20 system. The reference electrode was located between electrodes A1 and A2, and the earlobe was used as the reference ground of the electrode. The data sampling frequency was 256 Hz, and the hardware filter passband was 0.5 Hz~100 Hz. Every subject selected a median of 1 min of EEG data of acupuncture for the elimination of the effect of the insertion or withdrawal of needle and other possible factors. For signal preprocessing, the noise in the EEG data was filtered out to extract effective data with a band-pass finite impulse digital filter with a band pass frequency ranging from 0.5 Hz to 30 Hz. Then, systematic effects which might be caused by referencing to a particular channel were removed by referencing the EEG data of each channel to the average of all channels. The EEG data after preprocessing are shown in Figure 1c.

### 2.2. Measurement of Dimensionality

Dimensionality, the minimal number of dimensions necessary to offer a precise representation of neural activity, is defined as [38]:

(1) $$Dim\left(C\right)=\frac{{\left(\mathrm{Tr}\;C\right)}^{2}}{\mathrm{Tr}\;C^{2}}=\frac{{\left(\sum_{i}\lambda_{i}\right)}^{2}}{\sum_{i}\lambda_{i}^{2}},$$

where *C* is the covariance matrix of the activity vectors, and $\lambda_{i}$ is the *i*th eigenvalue of the covariance matrix *C*. In this work, *C* is the covariance matrix of the electrical signals of 19 electrodes. Dim(C)∈[1,19], where Dim(C)=19 indicates that the activity of the brain is independent and has equal variance, and Dim(C)=1 demonstrates strongly correlated brain activity.

### 2.3. Method for Extracting Low-Dimensional Latent Variables

**Figure 2:**
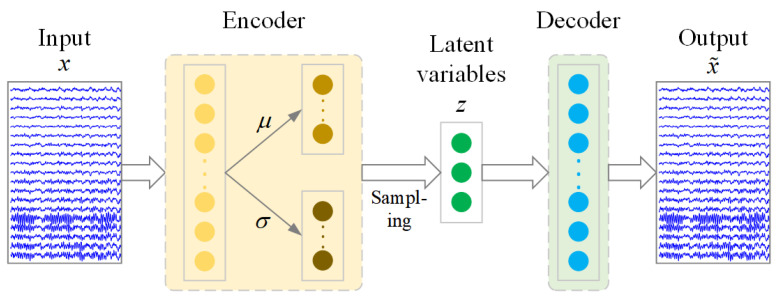
Neural network architectures of VAE. The encoder is in charge of encoding the high-dimensional input (*x*) into a low-dimensional representation (*z*), and the decoder is in charge of reestablishing the input data (*x*) on the basis of the low-dimensional representation (*z*).

The variational auto-encoder (VAE) is a powerful deep learning method for extracting the latent variables from data, which occurs in a feedforward manner, consisting of symmetrical networks: the “encoder” and “decoder” (as shown in Figure 2). More specifically, the encoder is in charge of encoding the high-dimensional input into a low-dimensional representation, and the decoder is in charge of reestablishing the input data on the basis of the low-dimensional representation.

Considering the dataset $\chi ={\left\{x\left(t\right)\right\}}_{t=1}^{N}$ of variable *x*, the VAE assumes that one random process involving an unobservable latent variable *z* generates all the data, which are produced from one prior distribution $p_{\theta }\left(z\right)$, thus *x* is determined by the conditional distribution $p_{\theta }\left(x|z\right)$ [35]. According to the Bayesian theory, the “decoder” network is in the form:

(2) $$x\sim p_{\theta }\left(x|z\right)p_{\theta }\left(z\right),$$

and the “encoder” network is of the form:

(3) $$p_{\theta }\left(z\right)\sim q_{\phi }\left(z|x\right)p\left(x\right).$$

The optimization function is defined based on minimizing the difference between the reconstructed data (output) and the original data (input), which is of the form:

(4) $$\max{Ε}_{q_{\phi }\left(z|x\right)}\left[\log p_{\theta }\left(x|z\right)\right]-D_{KL}\left(q_{\phi }\left(z|x\right)\|p_{\theta }\left(z\right)\right).$$

According to the Monte Carlo estimation method, the first term in the equation above is calculated through sampling *L* times as follows:

(5) $$E_{q_{\phi }\left(z|x\right)}\left[\log p_{\theta }\left(x|z\right)\right]=\frac{1}{L}\sum_{l=1}^{L}\log p_{\theta }\left(x\left(t\right)|z_{l}\left(t\right)\right)$$

The KL divergence of the approximate posterior $q_{\phi }\left(z|x\right)$ from the true prior $p_{\theta }\left(z\right)$ is computed through $-D_{KL}\left(q_{\phi }\left(z|x\right)\|p_{\theta }\left(z\right)\right)=\frac{1}{2}\sum_{j=1}^{J}\left(1+\log\left(\sigma_{j}^{2}\left(t\right)\right)-\mu_{j}^{2}\left(t\right)-\sigma_{j}^{2}\left(t\right)\right)$, where *J* is the dimensionality of *z*.

We utilized stochastic gradient descent and a back-propagation method to optimize the unknown parameter θ and the latent variable *z* by minimizing the difference between the output data and the input data. In this work, the 3-min-long dataset under different states was cut into 18 10-s-long data segments; thus, the number of samples for one segment is 2560. Hence, the batch size for unsupervised VAE learning is set as 20 to balance the training speed. The VAE approach was realized through the Deep Learning Toolbox in Matlab (R2021b).

## 3. Results

### 3.1. The Oscillatory Properties of Brain Activity Evoked via Manual Acupuncture Stimulation

**Figure 3:**
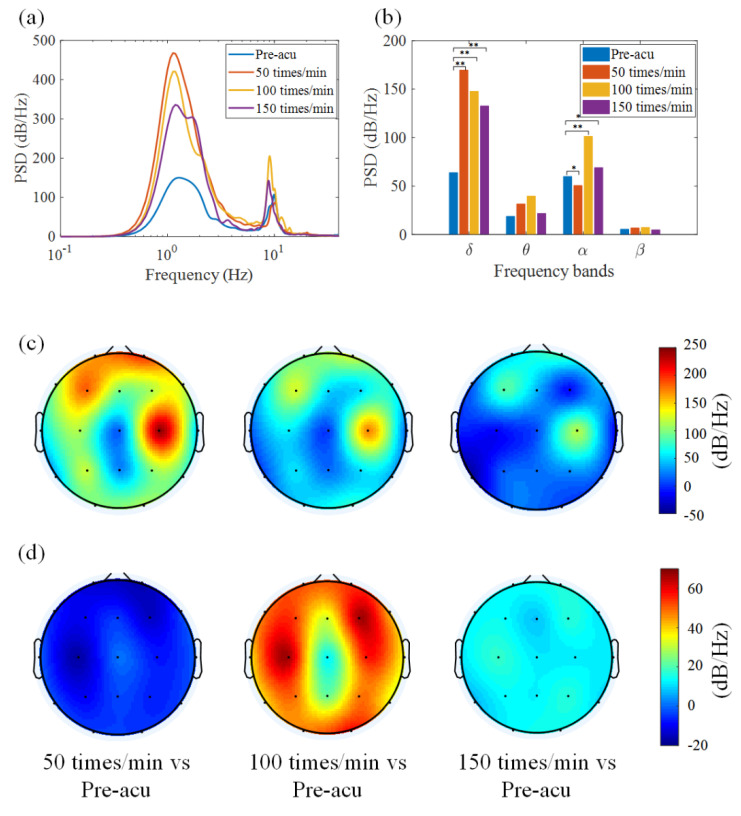
Brain activity associated with manual acupuncture stimulation. (**a**) Power spectrum char-acteristics of EEG data under different states. (**b**) PSD distribution in different frequency bands. *p* < 0.05 (*) and *p* < 0.01 (**) represent significant difference levels between pre-acupuncture and acu-puncture states. (**c**,**d**) Topographic map showing the variance of the PSD distribution between ac-tivity during and before different acupuncture manipulation states in (**c**) delta and (**d**) alpha fre-quency bands. Acupuncture can significantly affect the oscillatory activity in the delta and alpha frequency bands within EEGs. This variance is increased in the frontal and parietal lobes.

Brain activity is composed of high-dimensional complex oscillatory activity with rich rhythmic information. Therefore, the power spectrum density (PSD) of EEG signals was first investigated using the Welch method. Before acupuncture, the energy reaches two peaks near 1.2 Hz and 10 Hz, and the energy is mainly concentrated in the low-frequency band (1.2 Hz, the delta frequency band), as shown in Figure 3a. In the acupuncture state, the tendency of the energy distribution is similar to the pre-acupuncture state, but with a significant increment in energy in the delta and alpha frequency bands compared with the state before acupuncture. The results show that acupuncture at ST36 could affect the neural oscillatory activity, especially in the delta and alpha frequency bands.

We further computed the average energy distribution across four sub-bands (delta, theta, alpha and beta), as shown in Figure 3b. Particularly, the energy in the delta frequency band was higher when the manipulation frequency was 50 times/min and 100 times/min. This phenomenon implies the emergence of resonance induced by acupuncture. As shown in Figure 3a,b, the neural activity oscillates at an inherent frequency (about 1.2 Hz). When the frequency of external stimulation comes close to this inherent frequency, the phenomenon of resonance occurs; thus, the oscillatory response in the delta band is amplified. The results indicate that the neural system may encode and transmit the acupuncture stimulus through resonance. Scientific studies have documented the experimental occurrence of resonance in electrical processes of the human brain, as recorded by EEG, elicited by mechanical tactile stimuli [39]. It can be inferred that resonance is one of the mechanisms by which the neural system encodes acupuncture stimulation.

In order to investigate the resonance effect of acupuncture on neural oscillations across brain regions, we calculated the PSD variance (the difference in the PSD value between the acupuncture state and the pre-acupuncture state). Figure 3c,d present the PSD variance in two typical frequency bands (delta and alpha). In the delta frequency band, energy in the frontal and parietal lobes is increased, especially in the left frontal lobe and the right parietal lobe. In the alpha frequency band, the energy is increased under acupuncture stimulation, except for the manipulation at 50 times/min. The findings obtained here are consistent with other experimental reports based on fMRI and PET data. Xiang et al. found that the brain regions that responded to acupuncture at ST36 only (specifically) were the inferior parietal lobe, the middle inferior gyrus, the posterior lobe of cerebellum, and the angular gyrus [40].

### 3.2. Dimensionality of Brain Activity

**Figure 4:**
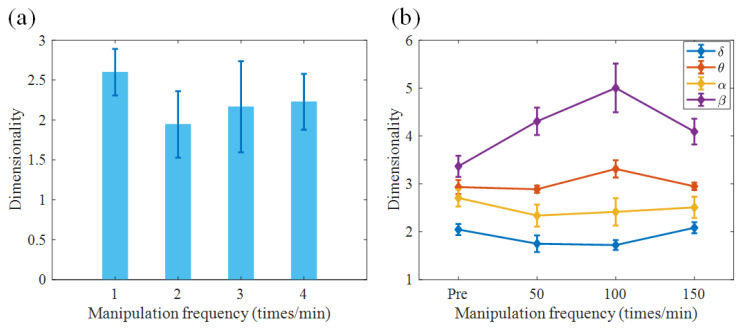
Dimensionality of brain activity. (**a**) Dependence of dimensionality of brain activity on acupuncture manipulation. (**b**) Dependence of dimensionality of brain activity in different sub-bands on acupuncture manipulation. Acupuncture can reduce the dimensionality of brain activity, especially with the manipulation at 50 times/min. The dimensionality in the delta and alpha frequency band was lower than that in the other two bands.

Recent research has investigated the dimensionality of neural ensembles from the sensory cortex of alert rats during periods of ongoing and stimulus-evoked activity, and found that stimuli could reduce the dimensionality of cortical activity [38]. Acupuncture is an external stimulation to the sensory system. It is of great importance to investigate whether the dimensionality of neural activity is affected by acupuncture. Figure 4a computes the dimensionality across all trials in the empirical dataset before and during acupuncture. The average dimensionality of brain activity in the pre-acupuncture state was larger than that in the acupuncture state. Moreover, the value of the dimensionality increased with an increase in the manipulation frequency. The dimensionality was minimal when the manipulation frequency was 50 times/min.

Furthermore, the dimensionality of neural activity in each sub-frequency band is explored in Figure 4b. The dimensionality in the delta and alpha frequency bands was smaller than that in the theta and beta frequency bands. In the delta and alpha frequency bands, the dimensionality was minimal when the manipulation frequency was 50 times/min, whereas in the theta and beta frequency bands, the dimensionality was maximized by acupuncture stimulation with a manipulation frequency of 100 times/min. Indeed, the oscillatory activity was more coherent in the delta and alpha frequency bands. It can be inferred that the enhancement of the correlated activity in the delta and alpha frequency bands induced by acupuncture could reduce the dimensionality of brain activity.

### 3.3. Low-Dimensional Dynamics of Brain Activity

Acupuncture’s effects are higher-order processes that are produced by the collaborative involvement of various latent brain factors, including different brain areas and physical or functional brain networks [41]. For example, Dhond et al. have confirmed that acupuncture may exert its therapeutic effects on pain by modulating a distributed network of brain areas involved in sensory, autonomic, and cognitive/affect processing, including endogenous antinociceptive limbic networks, as well as cognitive and affective control centers within the prefrontal cortex and the medial temporal lobe [10]. Moreover, the relationships between acupuncture analgesia and attentional mechanisms have been gradually revealed [42]. As EEG results are an external manifestation of the latent brain factors’ activities, it is of great importance to probe the low-dimensional dynamics of brain activity associated with acupuncture stimulation based on multichannel EEG signals.

**Figure 5:**
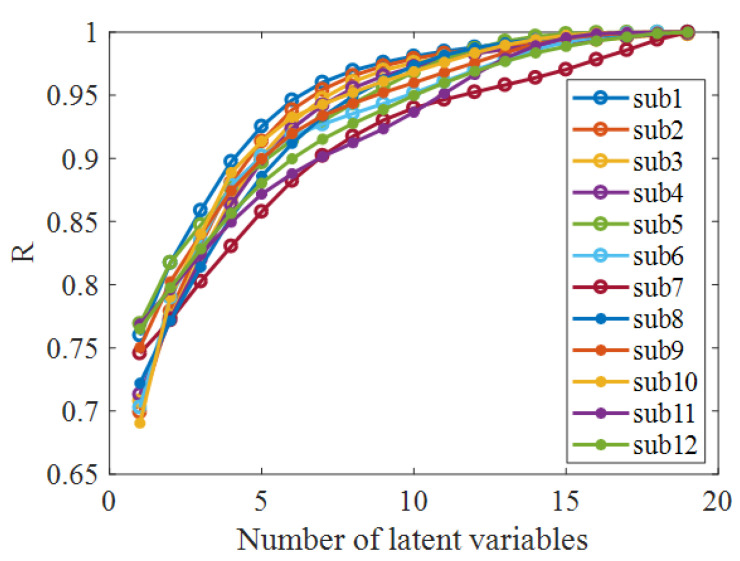
The reconstruction performance of VAE using different numbers of latent variables. The reconstruction performance increased with the enlargement of the latent variable number.

We employed the VAE method to extract the low-dimensional latent variables from the EEGs recorded before and during acupuncture. First, the reconstruction performance of VAE under different assumed numbers of latent variables was investigated. The reconstruction performance was quantified as the mean correlation between the original and reconstructed EEG channel signals. As shown in Figure 5, the performance gradually improved with an increasing number of latent variables for all subjects. When the number of latent variables was greater than three, the model was able to obtain a reconstruction performance of more than 80% on the EEG dataset.

**Figure 6:**
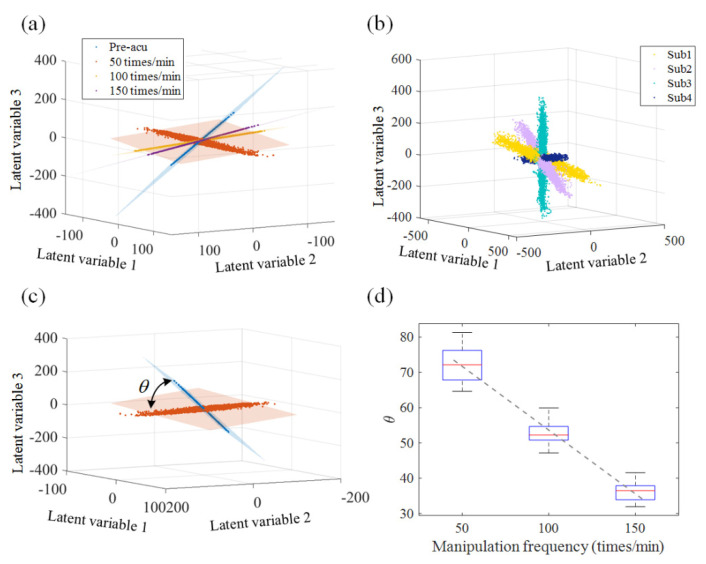
Latent variables in a 3-dimensional plane. (**a**) Different states of one subject. (**b**) Four ran-dom selected subjects in the pre-acupuncture period. Each color trace corresponds to a single trial. (**c**) Illustration of the variance of the latent dynamic space, where the angle between reference plane (pre-acupuncture state, orange) and acupuncture state (50 times/min, blue) was measured as θ. (**d**) Relationship between acupuncture manipulation and the plane included angle. Using VAE, the low-dimensional subspace of brain activity can be identified. The characteristics of the subspaces were determined by individuals and acupuncture stimulations.

We further examined the dynamic properties of these latent variables extracted from the EEG dataset. For each acupuncture stimulation, we plotted the top 3 dimensions of latent variables in Figure 6. It was shown that all units in each acupuncture manipulation operation contributed to a span, which is known as a latent dynamic space. Each latent dynamic space captured a population-wide activity pattern. For different subjects, the latent factors of different states still formed a latent dynamic space, but they had different planes (Figure 6b). To test whether the neural latent dynamic spaces corresponded to different manipulation frequencies, we set the latent dynamic space formatted by the pre-acupuncture period as the reference plane (or null plane), and computed the angles between each plane (induced by each different acupuncture stimulation) and the reference plane. The measurement is depicted in Figure 6c. The statistical results shown in Figure 6d demonstrate that although the planes of different individuals varied, the angles between them and the reference plane remained unchanged with different subjects. Moreover, the angle (θ) linearly depends on the manipulation frequency with a high goodness of fit of 0.78.

**Figure 7:**
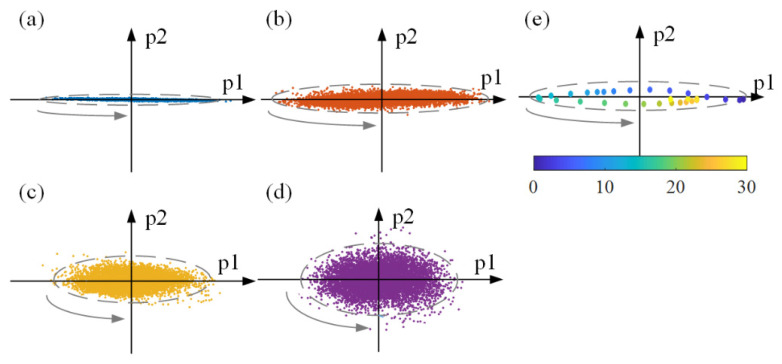
Schematic diagram of the trajectory under different acupuncture manipulation frequencies in latent dynamic space. (**a**) Pre-acupuncture, (**b**) 50 times/min, (**c**) 100 times/min, (**d**) 150 times/min. (**e**) An illustration exhibiting points’ evolution over time in (**b**). The color labels present the time order of each point, and the time step between points is 1/256 s. It can be seen that the units representing time-varying activity in the neural space converge to an elliptic attractor.

**Figure 8:**
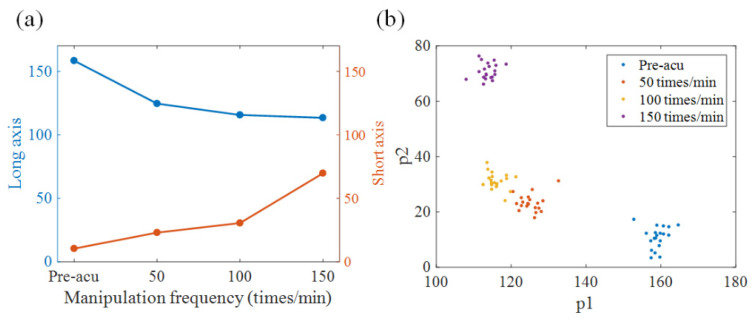
Statistical analysis of attractors of different states. (**a**) Dependence of long and short axes on manipulation frequency. (**b**) Cluster of manipulation operation based on attractors. The statistics of attractors can be discriminants for different brain states.

In addition, we inspected the dynamics of the top three latent variables in each latent dynamic space, as shown in Figure 7. It was evident that the units representing time-varying activity in the neural space converged to an ellipse (defined as an attractor). The trajectory was mostly confined to the latent neural space, a plane shown in Figure 7 and spanned by the acupuncture modes p1 and p2. The arrow in each figure reflected the direction of the trajectory as it evolved over time. Intuitively, the long axes of the elliptic attractor increased. We computed the mean distance of the long and short axes across different trials and plotted them in Figure 8a. The quantitative results confirmed that the variance trends were influenced by different acupuncture manipulations. A one-way analysis of variance (ANOVA) was applied to determine whether there were any statistically significant differences in the attractors between acupuncture states. The index *p* was calculated based on the mean and variance of the length of the long and short axes of the elliptic attractors in each state. Table 1 indicates that the long and short axes of the attractor in each state had significant differences, where *p* < 0.05 (*) and *p* < 0.01 (**) stand for their significance levels in statistical analysis. Furthermore, the difference between p1 and p2 was calculated between any two states in Table 2. The maximum *p*-value was on the order of 10^−3^, far less than 0.01. The obtained results confirmed the statistically significant differences of the attractors.

Based on the different statistical characteristics of the attractors, the neural dynamics of different trials induced by different acupuncture manipulation conditions were clustered (as shown in Figure 8b). In order to automatically classify different states, four machine learning models—a support vector machine (SVM), the k-nearest neighbor (KNN) method, linear discriminant analysis (LDA), and decision trees (DTs)—were constructed. The length of the long and short axes extracted from the low-dimensional attractors were considered for the training of the classifier model. The average accuracy of the acupuncture classification was calculated by means of five-fold cross validation, conducted 10 times. Table 3 compares the mean classification accuracy obtained for these machine learning models. It indicates that all these four models were able to achieve more than 95% classification accuracy. This result suggests the universality of the proposed classification scheme based on the statistical characteristics of the attractors. Furthermore, the performance of LDA was better than that of the other three classifiers.

## 4. Discussion

The present study was aimed at probing the low-dimensional dynamics of brain activity associated with acupuncture at the ST36 acupoint with different manipulation frequencies. Specifically, we studied the changes in the power spectrum of brain activity before and during acupuncture stimulation. We extracted the neural subspace and characterized the relationship between acupuncture stimuli and low-dimensional dynamics.

Using a manual acupuncture paradigm, in conjunction with brain electroencephalography (EEG) signal recording, we observed that acupuncture episodes were associated with increased spectral power in the delta and alpha frequency bands compared to episodes of resting, especially in the delta frequency band. This phenomenon suggests that stochastic resonance is a way in which the brain processes periodic acupuncture stimulation. Stochastic resonance is commonly understood to be the enhancement of the response of a nonlinear system in cases where the frequency of the external input is close to its intrinsic oscillatory frequency, with the help of noise [43,44]. Noise, which is ubiquitous in the brain, comes from synaptic transmission, channel gating, ion concentrations, and membrane conductance, and is possibly involved in stochastic resonance phenomena [45,46]. In the acupuncture experiment, when the stimulation frequency was close to the intrinsic frequency of the cerebral oscillations (the delta frequency band), the rhythmic activity of the cerebral oscillation was enhanced. This enhancement was mainly concentrated in the parietal lobe, which is associated with the somatosensory area. Resonance in the central nervous system of mammalians may account for their higher brain functions, such as human tactile sensations, visual perception, and animal feeding behavior [47,48]. In this study, we preliminarily found a resonant response of the brain to acupuncture stimulation. More experimental and analytical studies will be carried out to investigate the potential benefits of stochastic resonance in acupuncture information processing in the neural system.

Additionally, we found that acupuncture stimuli could reduce the dimensionality of the neural electrical response of the cerebral cortex. At present, the study of dimensionality in neural systems has attracted extensive attention [49,50,51]. Dimensionality analysis has been employed for various tasks and across neural systems [31,52]. For example, Rigotti et al. studied the relationship between the dimensionality of an evoked activity and task complexity, and suggested that the evoked dimensionality roughly amounted to the number of task conditions [53]. Acupuncture is a complex stimulation comprising multimodal sensory stimulations, including temperature, pressure, and noxious stimulations. Different manual acupuncture manipulations, such as lifting, thrusting, and twisting, contain different stimulating parameters, thus generating different responses to acupuncture [54]. The study of the dimensionality of brain activity under acupuncture stimuli will help to reveal the mechanisms underlying different acupuncture manipulations. Setting up an accurate experimental and theoretical connection between dimensionality and acupuncture manipulations, supported by an understanding of neural activity, is a significant question for further studies.

In this work, VAE was an efficient approach for reducing dimensionality and extracting latent variables from multichannel EEG signals. Essentially, the VAE adopted in this work was carried out in a feedforward manner, and this oversimplification of the network structure may result in lower effectiveness of VAE when the input becomes complex. One possible solution to this problem is to combine the recurrent network and VAE frameworks, which has been gradually applied in research on image recognition. In addition, the small world is a type of recurrent network with a smaller average transmission delay and more robust network connectivity. The combination of a small world network and the VAE framework may improve the processing performance for high-dimensional complex datasets and reduce the training time required.

Furthermore, using a dimensionality reduction method, we obtained a neural subspace of brain activity and found that the low-dimensional dynamics converged to topologically similar elliptic-shaped attractors. The brain state (pre-acupuncture or undergoing acupuncture with different manipulation frequencies) can be well classified based on the statistical characteristics of these attractors. The elliptical attractors implied characteristics of continuous fluctuation of the brain, which may result from internal variability (noise) and external stimuli. In a previous study [55], we observed fluctuations in the scaling of neural activity in a spontaneously active brain circuit. Olguin-Rodriguez et al. have investigated characteristic fluctuations around stable attractor dynamics extracted from highly nonstationary EEG recordings [56]. On the other hand, researchers have demonstrated that the dynamical regime of the sensory cortex converges to stable dynamics around a single stimulus-tuned attractor [57]. The attractor dynamics are not only associated with the properties of stimuli, but are associated with brain function. Finkelstein et al. showed that communication between brain regions can be gated via attractor dynamics, which control the degree of commitment to an action [58]. Therefore, it is of great importance to investigate the attractor dynamics of brain activity evoked by acupuncture stimuli, which will shed light on revealing the action mechanism of acupuncture.

Typical neural responses are shaped both by internal dynamics and various external stimuli. Even when exposed to the same external stimulation, different subjects responded differently, as their inherent internal dynamics are not quite the same. Consequently, the characteristics of low-dimensional dynamics extracted from multichannel EEG signals vary between individuals. Although differences between subjects and latent variables are informative for classification, there is still a key limitation of the proposed method, in that it cannot directly extract the stimulus-related variables from neural responses. Acupuncture can be regarded as a specific somatosensory stimulation on the acupoint, and can mediate the function of the human body via the nervous system. Furthermore, the VAE method neglects information about the relevant experimentally controlled variables. Therefore, in order to better probe the relationship between brain activity and acupuncture stimulation, we will decompose the acupuncture-evoked information from EEG signals, and further characterize the low-dimensional dynamics of acupuncture-evoked signals in the next step of our research. This further research will help to reveal the essential role of acupuncture.

As a complementary therapeutic treatment, acupuncture could improve symptoms in various neural diseases, such as depression, stroke rehabilitation, and Parkinson’s disease [59,60,61]. Increasingly, clinical experiments have shown that the effectiveness of acupuncture is related to changes in brain activity. For example, Chae et al. documented a significant improvement in the motor function of PD patients after acupuncture treatment. The putamen and the primary motor cortex were activated when patients with PD received acupuncture treatment and these activations correlated with individual enhanced motor function [62]. Moreover, it was found that acupuncture can reduce drug addiction via direct activation of brain pathways [63]. In this work, we confirmed that acupuncture can affect the characteristics of the latent neural subspace. For different neural diseases, we proposed that abnormal brain activity may be reflected by the characteristics of this subspace as well. In future works, we will conduct further clinical experiments to validate the relationship between these latent neural dynamics and the therapeutic effects of acupuncture. These results can provide a theoretical support for the selection of appropriate acupuncture frequencies for patients in clinical settings, and the proposed methods have potential in relation to exploring the effects of acupuncture on brain activity.

## 5. Conclusions

In this work, the low-dimensional dynamics of brain activity associated with acupuncture stimuli was probed. We found that manual acupuncture stimuli can reduce the dimensionality of brain activity, which results from the enhancement of oscillatory activity in the delta and alpha frequency bands induced by acupuncture. Moreover, it was found that large-scale brain activity could be approximated through the dynamics of a relatively simple attractor contained within a low-dimensional neural space, and the attractor’s morphology was closely related to the frequency of acupuncture stimulation. These results shed light on the large-scale brain response to manual acupuncture stimuli.

## Figures and Tables

**Table 1:** ANOVA 1 analysis for comparison of the length of the long and short axes in different states.

Axis	Pre-Acu	50 Times/Min	100 Times/Min	150 Times/Min
p1 vs. p2	$1.65\times 10^{-33}$	$5.08\times 10^{-29}$	$1.00\times 10^{-27}$	$3.07\times 10^{-17}$

**Table 2:** ANOVA 1 analysis for comparison of the length of the long and short axes in different states, respectively.

Axis	Pre-Acu vs. 50 Times/Min	Pre-Acu vs. 100 Times/Min	Pre-Acu vs. 150 Times/Min	50 Times/Min vs. 100 Times/Min	50 Times/Min vs. 150 Times/Min	100 Times/Min vs. 150 Times/Min
p1	$8.05\times 10^{-13}$	$1.89\times 10^{-28}$	$1.46\times 10^{-29}$	$1.28\times 10^{-3}$	$8.28\times 10^{-7}$	$2.61\times 10^{-3}$
p2	$1.34\times 10^{-3}$	$1.72\times 10^{-10}$	$7.20\times 10^{-28}$	$8.52\times 10^{-3}$	$2.03\times 10^{-26}$	$9.15\times 10^{-24}$

**Table 3:** Mean classification accuracy for various machine learning models.

Model	SVM	KNN	LDA	DT
Accuracy	97.5%	97.5%	98.8%	95.0%

## Data Availability

Not applicable.
